# Atypical Presentation of Primary Hyperparathyroidism

**DOI:** 10.7759/cureus.74337

**Published:** 2024-11-24

**Authors:** Win Thu, Aisha Hashmi

**Affiliations:** 1 Diabetes and Endocrinology, University Hospitals Plymouth National Health Service (NHS) Trust, Plymouth, GBR

**Keywords:** acute psychosis, adult primary hyperparathyroidism, first episode psychosis, hypercalcemia, parathyroid gland adenoma

## Abstract

Primary hyperparathyroidism (PHPT) typically presents with a spectrum of symptoms, including neuropsychiatric manifestations such as anxiety, depression, confusion, and, in severe cases, coma. While psychiatric symptoms are not uncommon in PHPT, acute psychosis is a rare presentation. In such cases, immediate control of serum calcium levels is crucial, and emergency parathyroidectomy may be required if medical management alone fails to control hypercalcemia.

A middle-aged female was initially evaluated in the endocrine clinic for newly diagnosed asymptomatic PHPT and was scheduled for elective parathyroidectomy. However, she subsequently presented to the hospital with acute psychosis and elevated serum calcium levels. Despite aggressive intravenous fluid resuscitation and bisphosphonate therapy, her calcium levels and psychiatric symptoms did not improve. She underwent an emergency parathyroidectomy, during which three parathyroid adenomas were identified and removed. Postoperatively, her psychosis resolved, and her serum calcium levels normalized. Genetic testing later ruled out Multiple Endocrine Neoplasia (MEN) syndrome.

Multiple parathyroid adenomas represent a rare but important subset of primary hyperparathyroidism. In cases with unusual presentations such as acute psychosis, prompt surgical intervention is essential, as it can lead to the rapid resolution of both hypercalcemia and neuropsychiatric symptoms. Genetic testing for MEN syndromes should be considered in cases of multiple adenomas to ensure appropriate follow-up and management.

## Introduction

Primary hyperparathyroidism (PHPT) is typically a benign condition, often presenting with asymptomatic hypercalcemia, which is detected incidentally during routine blood tests. However, it can manifest with a spectrum of symptoms, ranging from mild gastrointestinal disturbances such as anorexia, nausea, and constipation to severe neuropsychiatric dysfunction, including confusion and coma. Psychiatric manifestations, although not uncommon in PHPT, rarely escalate to acute psychosis, making such presentations unusual. 

While solitary parathyroid adenomas account for approximately 90% of PHPT cases, multiple parathyroid adenomas are rare, occurring in only 2-11% of cases [1]. In this report, we present a unique case of PHPT presenting as acute psychosis caused by multiple parathyroid adenomas, for which the patient underwent urgent removal of the affected parathyroid gland. This is curative in most cases, resulting in postoperative normalization of calcium and typically offering lifelong resolution [2].

## Case presentation

A 58-year-old female patient was referred by her general practitioner (GP) to the endocrinology outpatient clinic as her routine blood tests showed high calcium and further investigation with the GP revealed a high parathyroid level. She was asymptomatic at that time. ON assessment during her endocrine clinic appointment, the patient reported mild symptoms of hypercalcemia, including muscle aches and joint pain. But she denied other symptoms of hypercalcemia such as abdominal pain, anxiety, or depression. She has no previous history of urinary stones or family history of endocrine problems. Her DEXA (Dual-Energy X-ray Absorptiometry) scan was normal, and her renal ultrasound showed no evidence of kidney stones. She was advised to maintain adequate hydration with a daily fluid intake of 2-4 liters and was placed on the waiting list for elective parathyroidectomy. Laboratory tests revealed a parathyroid hormone (PTH) level of 25.1 pmol/L and a serum calcium level of 2.83 mmol/L. Further investigation, including a 24-hour urinary calcium-to-creatinine ratio of 0.0265, excluded a diagnosis of familial hypocalciuric hypercalcemia (FHH).

**Figure 1:**
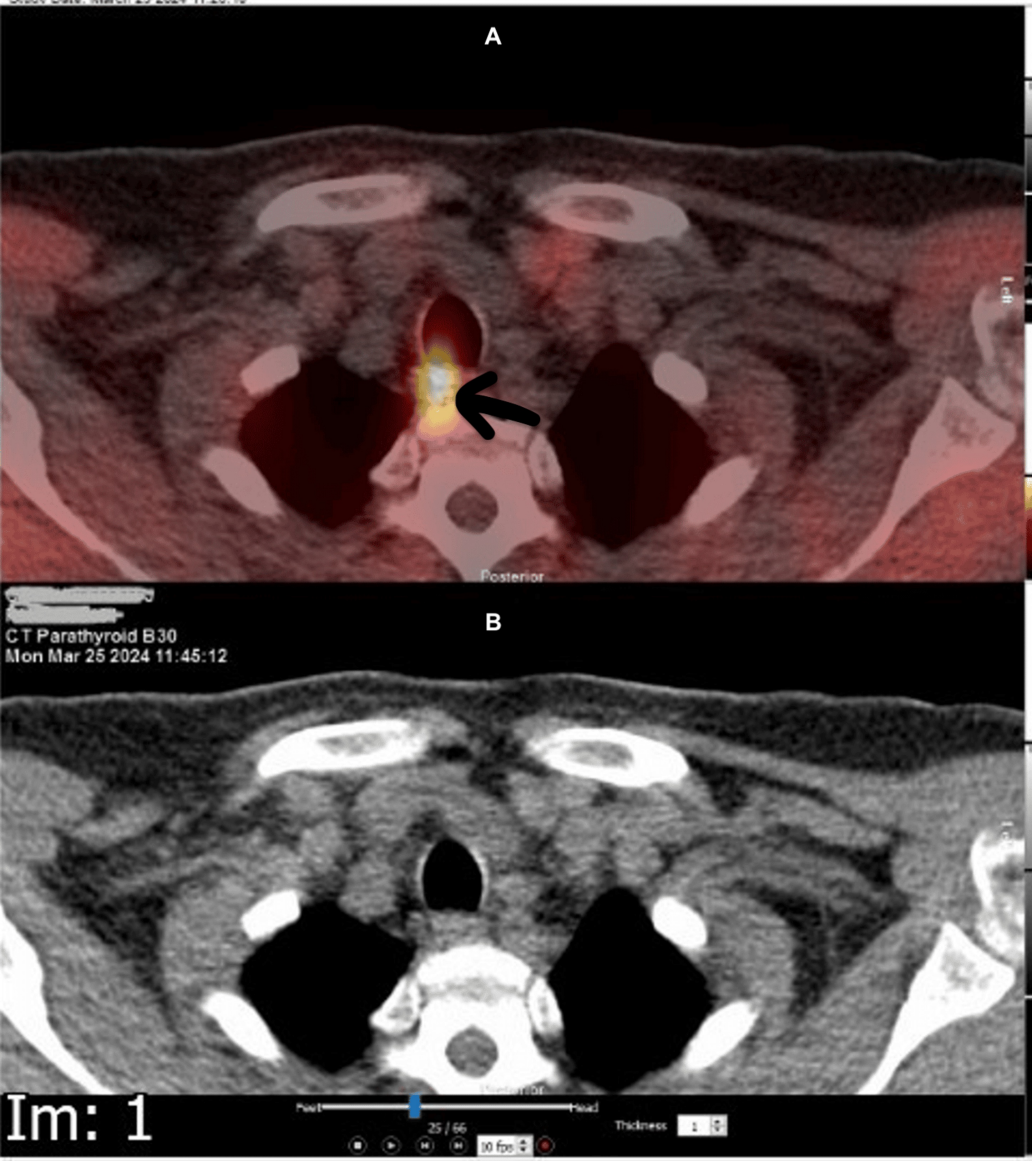
Sestamibi scan showing a small retro-tracheal focus on the right.

**Figure 2:**
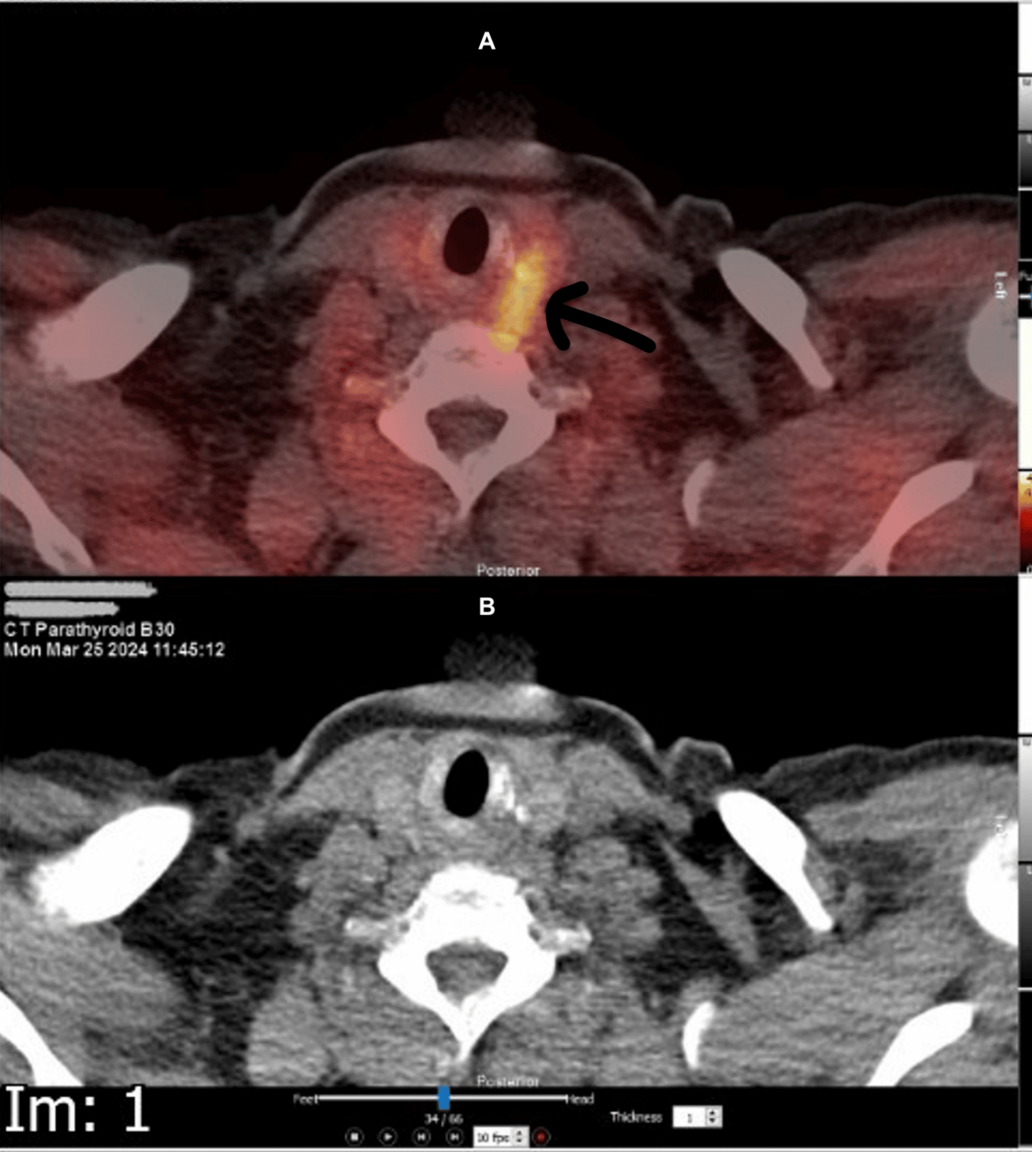
Sestamibi scan showing is residual tracer retention on left side and 11 mm soft tissue nodule posterior to the mid-portion of the left thyroid lobe.

Imaging studies included a sestamibi (technetium-99m sestamibi) scan, which demonstrated a small retro-tracheal focus on the right side (Figure 1) and an 11 mm soft tissue nodule posterior to the mid portion of the left thyroid lobe (Figure 2), suggesting bilateral parathyroid adenomas.

**Figure 3:**
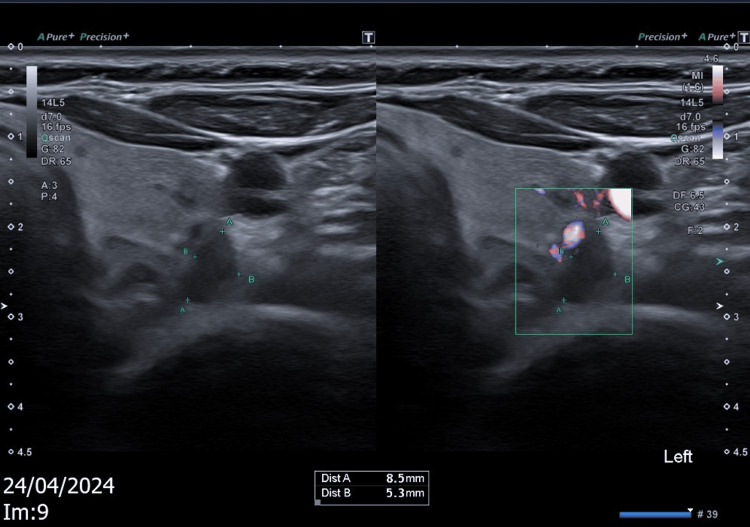
Neck ultrasound showing 9 mm hypoechoic lesion posterior to the lower pole of the left thyroid.

A neck ultrasound (US) did not reveal any abnormalities in the right tracheoesophageal groove but identified a 9 mm hypoechoic lesion posterior to the lower pole of the left thyroid, correlating with the sestamibi scan findings (Figure 3).

Three months later, while awaiting her appointment with the Ear Nose, and Throat (ENT) surgeon, she was admitted to the hospital following a sudden onset of worsening anxiety and marked behavioral changes. Notably, she exhibited an episode of acute psychosis, during which she attempted to suffocate her daughter with a pillow. Laboratory tests revealed a serum calcium level of 3.22 mmol/L and a PTH level of 20.8 pmol/L (Table 1). She was treated with aggressive intravenous (IV) fluid hydration and IV bisphosphonate therapy; however, her calcium levels remained elevated at 2.94 mmol/L, and her psychotic symptoms persisted. 

**Table 1:** Laboratory investigations.

Investigations	Results at clinic	Results at admission	Results after surgery	Normal reference range	Unit
Corrected calcium	2.83	3.22	2.27	2.2-2.6	mmol/l
Phosphate	0.5	0.7	1.1	0.81-1.45	mmol/l
Sodium	137	135	138	135-145	mmol/l
Potassium	4.5	4.3	3.9	3.5-5.5	mmol/l
Creatinine	48	50	52	53-97.2	umol/l
Glomerular filtration rate	>90	>90	>90		ml/min/1.7m^2
Parathyroid hormone	25.1	20.8	0.6	1.6-6.9	pmol/l
Vitamin D	45	42		<30: deficient, 30-50: inadequate, 50-75: adequate, >75: optimum	nmol/l
Calcium-creatinine clearance ratio	0.02652			<0.14	
White cell	12.1	13.5		4.5-11	10x9/l
Hemoglobin	146	149		121-151	g/l
Platelets	293	380		150-450	10x9/l
C-reactive protein		2		<10	mg/l
Genetic testing for MEN1, RET gene			negative		

Given her refractory hypercalcemia and severe neuropsychiatric symptoms, she underwent urgent parathyroidectomy, during which three glands (left inferior, left superior, and right superior parathyroid glands) were removed. The pathology report revealed adenoma of all three glands. Postoperatively, she recovered well, and her calcium levels normalized. Genetic testing for Multiple Endocrine Neoplasia (MEN) types 1 and 2A was negative. Since surgery, she has maintained normal serum calcium levels and has remained asymptomatic.

## Discussion

Neuropsychiatric disturbances are commonly observed in patients with hypercalcaemia, particularly those with primary hyperparathyroidism (PHPT). The most frequent symptoms include anxiety, depression, and cognitive dysfunction. In more severe cases, symptoms such as lethargy, confusion, stupor, and even coma may occur. However, acute psychosis as the initial manifestation of PHPT is exceedingly rare. 

Calcium is an essential ion in the body that plays a crucial role in various cellular processes, including neurotransmitter release, cellular signaling, and muscle contraction. When calcium levels become too elevated, it can disrupt these processes, particularly in the brain, leading to cognitive and psychiatric symptoms, including psychosis. Calcium ions are important for synaptic transmission and neuronal firing. In neurons, calcium enters cells through voltage-gated calcium channels and acts as a secondary messenger in signaling pathways. Elevated calcium levels can lead to abnormal neuronal excitability and synaptic dysfunction. Elevated calcium levels may cause glutamatergic excitotoxicity, along with disruptions in dopaminergic and serotonergic signaling pathways. Glutamatergic excitotoxicity is a process in which nerve cells are damaged and killed due to the excessive activation of glutamate receptors, particularly in the central nervous system (CNS). Glutamate is the main excitatory neurotransmitter in the brain and is involved in synaptic transmission, learning, and memory. However, when glutamate is released in excessive amounts, it can overactivated glutamate receptors, leading to toxic effects that contribute to neuronal injury and death [3]. Restoring normal calcium levels through surgical removal of the responsible parathyroid adenoma has been shown to rapidly alleviate neuropsychiatric symptoms. 

In most cases of PHPT, a single parathyroid adenoma is identified. However, in this case, we observed multiple adenomas affecting the left inferior, left superior, and right superior parathyroid glands. The pathogenesis of multiple parathyroid adenomas is not well understood, and there is no established correlation between the severity of psychosis and either the degree of hypercalcemia or the number of adenomas. This adds to the complexity of the clinical presentation and management. 

Imaging plays a crucial role in the preoperative identification of parathyroid adenomas. In our patient, the Sestamibi scan identified two adenomas, but it failed to definitively localize all three, making lateralization and surgical planning more challenging. Incomplete localization often results in longer operative times and may necessitate a more extensive neck exploration. More sensitive imaging techniques, such as four-dimensional computed tomography (4D-CT), could enhance the accuracy of adenoma detection, potentially shortening operative time and reducing the need for extensive surgery [4]. Further studies are warranted to evaluate the role of 4D-CT in the preoperative workup of PHPT, as its use is not yet widespread in many institutions. 

Additionally, although multiple parathyroid adenomas can sometimes be associated with genetic syndromes such as MEN, our patient tested negative for MEN types 1 and 2A, ruling out a syndromic cause in this case. MEN syndromes, particularly MEN type 1, are characterized by multiple endocrine tumors, including parathyroid hyperplasia or multiple adenomas, and thus genetic testing is critical in such cases to guide future surveillance and management. 

In conclusion, multiple parathyroid adenomas represent a rare but important subset of primary hyperparathyroidism. In cases with unusual presentations such as acute psychosis, prompt surgical intervention is essential, as it can lead to the rapid resolution of both hypercalcemia and neuropsychiatric symptoms. Genetic testing for MEN syndromes should be considered in cases of multiple adenomas to ensure appropriate follow-up and management.

## Conclusions

This case highlights the importance of considering primary hyperparathyroidism as a potential cause of acute psychosis, even though it is a rare presentation. Prompt recognition and management of severe hypercalcemia are critical, particularly when neuropsychiatric symptoms are prominent. In cases where medical management fails to control hypercalcemia, emergency parathyroidectomy can provide rapid resolution of symptoms. While primary hyperparathyroidism is commonly due to a single adenoma, the presence of multiple parathyroid adenomas, as observed in this case, underscores the need for careful diagnostic workup and surgical planning. Standard imaging may not always detect all adenomas preoperatively, which can complicate localization and increase operative time. More advanced imaging techniques, such as four-dimensional computed tomography (CT) scans, may improve detection rates and streamline surgical intervention in complex cases.

Furthermore, although our patient tested negative for Multiple Endocrine Neoplasia (MEN) types 1 and 2A, genetic testing should be considered in similar cases to guide future management and surveillance. This case underscores the need for awareness of atypical presentations of primary hyperparathyroidism and highlights the value of a comprehensive diagnostic and therapeutic approach to achieve optimal patient outcomes.
